# Supplementary material for: Six groups of ground-dwelling arthropods show different diversity responses along elevational gradients in the Swiss Alps
Source: PLoS One. 2022 Jul 25;17(7):e0271831. doi: 10.1371/journal.pone.0271831 (PMC9312367; doi:10.1371/journal.pone.0271831)
Supplement: S1 File — (DOCX) [file pone.0271831.s001.docx]

**S1 File. Methodology, nomenclature and literature used for species identification**

In all arthropod groups the specimens captured were identified to species level.

Spiders were identified using the keys of Nentwig et al. (2020). The nomenclature followed World Spider Catalog (2020).

Lithobiomorph centipedes were identified following Eason (1982), complemented with updated checklists and taxonomic works (e.g. Stöckli 2009). Geophilomorph centipedes were identified following ChiloKey (Bonato et al. 2014). All species identifications were subsequently confirmed by comparing the specimens with the original descriptions of the species. The nomenclature followed Chilobase (Bonato et al. 2016), except in cases when more recent taxonomic works proposed a different name or synonymy.

Millipede specimens were identified by comparing external and gonopod morphology (modified copulatory 8th leg of the male), in some cases after dissection, with either the descriptions and drawings in Bigler (1929) or the original descriptions of the species. In the case of the genus *Glomeris* Latreille, 1802, identifications followed the keys and drawings in Hoess (2000). When the original description was not accessible, specimens were compared to individuals from the collection of the Naturhistorisches Museum Basel. The nomenclature followed Kime & Enghoff (2011, 2017) for the orders Glomerida, Polydesmida and Julida, and the Fauna Europaea website (Enghoff 2017) for the order Chordeumatida.

Ants were identified using the keys in Seifert (2007). Where necessary keys and trait tables in recent taxonomical papers were used additionally (e.g. Seifert 2021, Seifert & Schultz 2021 for some species in the genus *Formica*). Nomenclature followed antwiki.org.

Ground beetles (Carabidae) were identified following Freude et al. (2006). Most of the specimens could be identified by their external morphology, but in some cases the examination of the aedeagus was necessary. The nomenclature follows the Catalogue of Palaearctic Coleoptera (Löbl & Löbl 2017).

Rove beetles (Staphlinidae) were determined using Freude et al. (1974, 2012). The nomenclature followed Freude et al. (1974, 2012), except in cases in which more recent taxonomic work was available.

The majority of the collected arthropods are deposited in the Bündner Naturmuseum in Chur (Grisons).

**References for S1 File**

Antwiki. 2020. Available at http://www.antwiki.org/Main_Page

Bigler W. 1929. Die Diplopodenfauna des Schweizerischen Nationalparks. *Ergebnisse der wissenschaftlicher Untersuchungen des Schweizerischen Nationalparks* 5: 1–87.

Bonato L, Chagas Junior A, Edgecombe GD, Lewis JGE, Minelli A, Pereira LA, Shelley RM, Stoev P, Zapparoli M. 2016. *ChiloBase 2.0 − A World Catalogue of Centipedes (Chilopoda)*. Available at https://chilobase.biologia.unipd.it.

Bonato L, Minelli A, Lopresti M, Cerretti P. 2014. ChiloKey, an interactive identification tool for the geophilomorph centipedes of Europe (Chilopoda, Geophilomorpha). *ZooKeys* 443: 1−9.

Eason EH. 1982. A review of the north-west European species of Lithobiomorpha with a revised key to their identification. *Zoological Journal of the Linnean Society* 74: 9−33.

Enghoff H. (group coordinator). 2017. *Diplopoda. Fauna Europaea version 2017.06*. Available at https://fauna-eu.org

Freude H, Harde KW, Lohse GA. 1974. *Die Käfer Mitteleuropas. Bd. 5, Staphylinidae 2 (Hypocyphtinae und Aleocharinae); Pselaphidae*. Goecke & Evers, Krefeld, Germany, 381 pp.

Freude H, Harde KW, Lohse, GA. 2012. *Die Käfer Mitteleuropas. Bd. 4, Staphylinidae (exklusive Aleocharinae, Pselaphinae und Scydmaeninae)*. Spektrum Akademischer Verlag, Heidelberg, Germany, 572 pp.

Freude H, Harde KW, Lohse GA, Klausnitzer B. (eds). 2006. *Die Käfer Mitteleuropas. Band 2. Adephaga 1: Carabidae (Laufkäfer), 2. Auflage*. Spektrum Akademischer Verlag, Heidelberg/Berlin, Germany, 521 pp.

Hoess R. 2000. Bestimmungsschlüssel für die Glomeris-Arten Mitteleuropas und angrenzender Gebiete (Diplopoda: Glomeridae). *Jahrbuch des Naturhistorischen Museums Bern* 13: 3–20.

Kime R.D. & Enghoff H. 2011. *Atlas of European Millipedes (Class Diplopoda): Volume 1, Orders Polyxenida, Glomerida, Platydesmida, Siphonocryptidae, Polyzoniida, Callipodida, Polydesmida*. Pensoft, Sofia-Moscow, 282 pp.

Kime R.D. & Enghoff H. 2017. Atlas of European millipedes 2: Order Julida (Class Diplopoda). *European Journal of Taxonomy* 346: 1–299.

Löbl I, Löbl D. (eds). 2017. *Catalogue of Palaearctic Coleoptera, Archostemata – Myxophaga –Adephaga, Revised and Updated Edition, Volume 1*. Brill, Leiden, Boston, 1443 pp.

Nentwig W, Blick T, Bosmans R, Gloor D, Hänggi A, Kropf C.2020. *Spiders of Europe. Version 5.2020*. Available at https://www.araneae.nmbe.ch. https://doi.org/10.24436/1

Seifert B. 2007. *Die Ameisen Mittel- und Nordeuropas*. Tauer: lutra-Verlags- und Vertriebsgesellschaft, 368 pp.

Seifert B. 2021. A taxonomic revision of the Palaearctic members of the Formica rufa group (Hymenoptera: Formicidae) – the famous mound-building red wood ants. *Myrmecological News* 31: 133–179.

Seifert B, Schultz R. 2021. A taxonomic revision of the Palaearctic ant subgenus Coptoformica Müller, 1923 (Hymenoptera, Formicidae). *Contributions to Entomology* 71: 177–220.

Stöckli E. 2009. Literature-based survey on the Swiss fauna of Chilopoda. *Soil Organisms* 81: 647–669.

World Spider Catalog (2020). *World Spider Catalog. Version 21.5*. Natural History Museum Bern. Available at http://wsc.nmbe.ch
