# Supplementary figures and images for: Six groups of ground-dwelling arthropods show different diversity responses along elevational gradients in the Swiss Alps
Source: PLoS One. 2022 Jul 25;17(7):e0271831. doi: 10.1371/journal.pone.0271831 (PMC9312367; doi:10.1371/journal.pone.0271831)

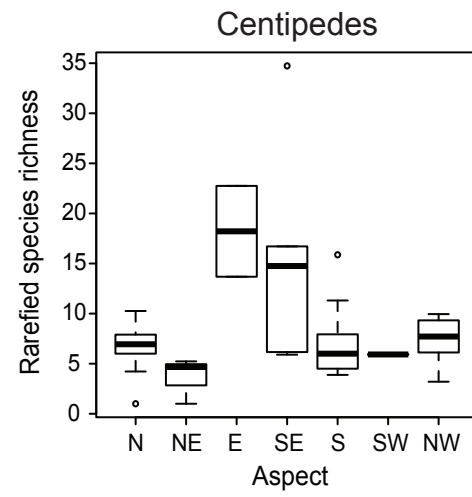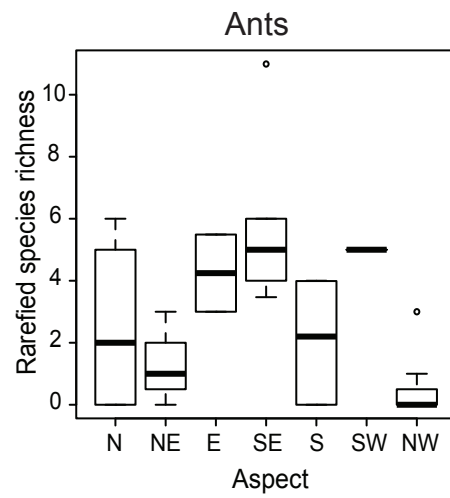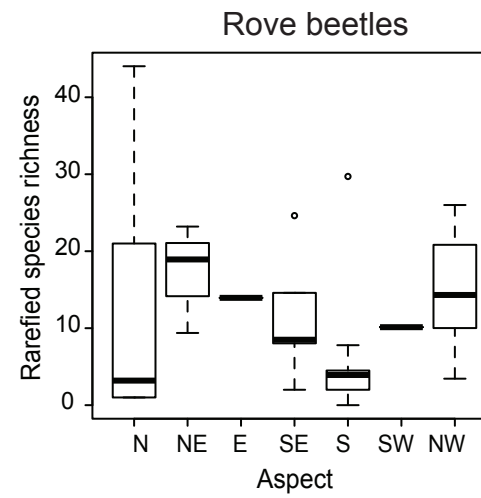

Supplement: S1 Fig — (PDF) [file pone.0271831.s002.pdf]

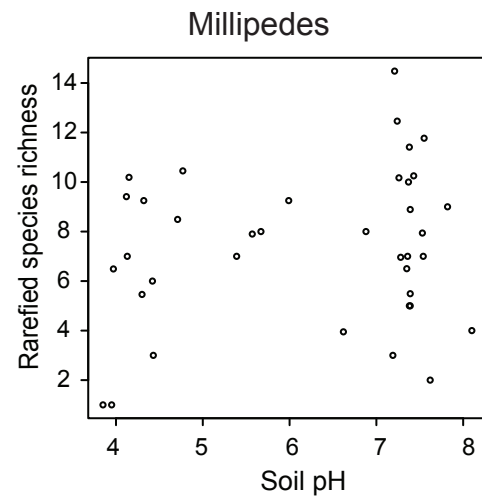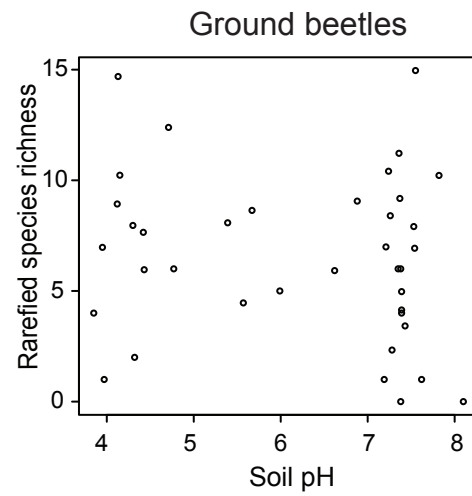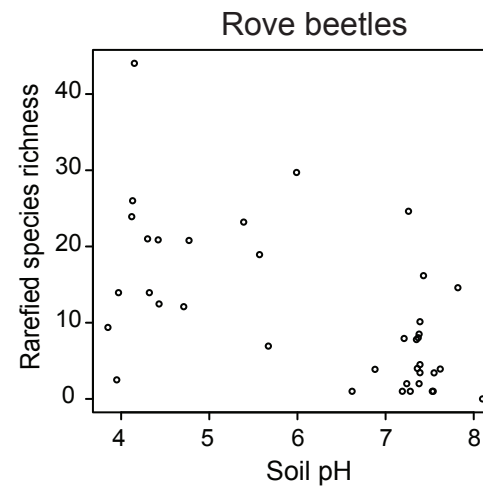

Supplement: S2 Fig — (PDF) [file pone.0271831.s003.pdf]

### Millipedes

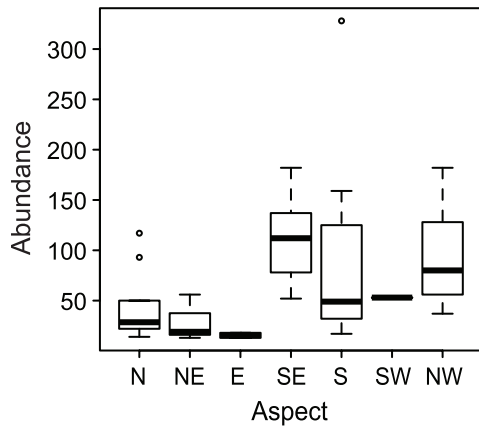

### Ants

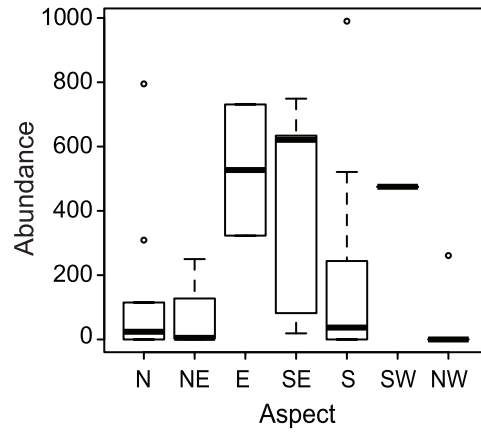

### Rove beetles

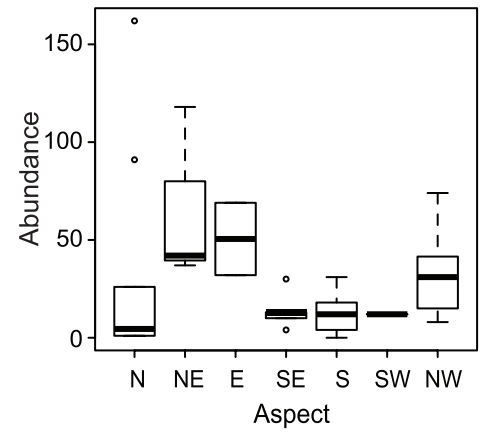

Supplement: S3 Fig — (PDF) [file pone.0271831.s004.pdf]

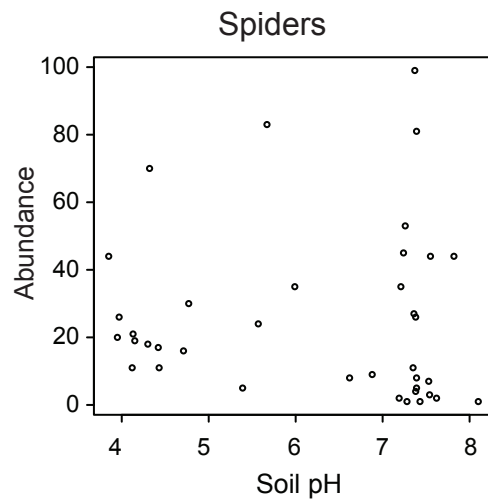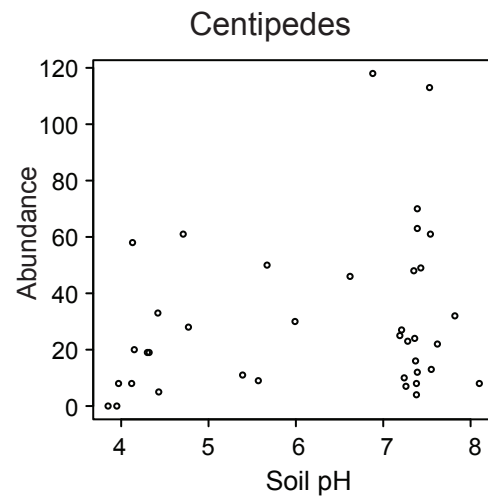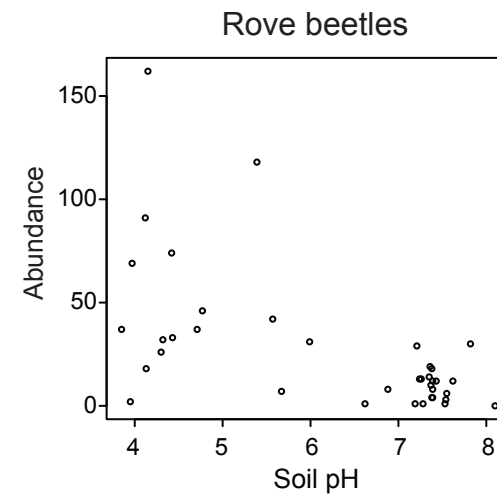

Supplement: S4 Fig — (PDF) [file pone.0271831.s005.pdf]

### Spiders

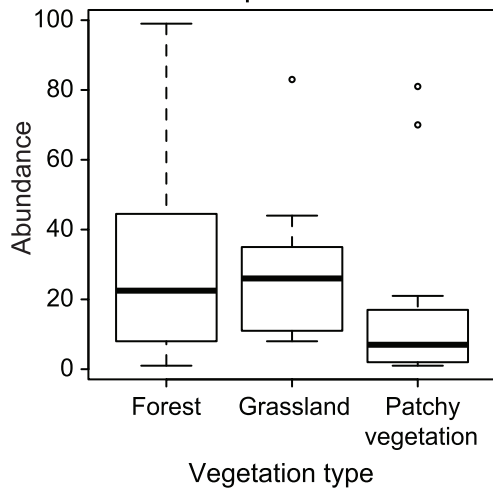

### Ground beetles

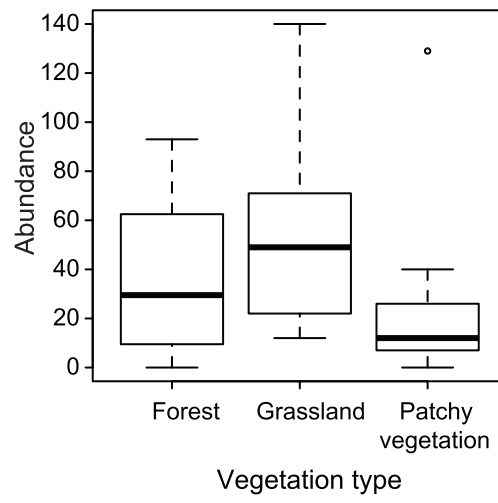

Supplement: S5 Fig — (PDF) [file pone.0271831.s006.pdf]
