# Supplementary material for: Six groups of ground-dwelling arthropods show different diversity responses along elevational gradients in the Swiss Alps
Source: PLoS One. 2022 Jul 25;17(7):e0271831. doi: 10.1371/journal.pone.0271831 (PMC9312367; doi:10.1371/journal.pone.0271831)
Supplement: S3 Table — B − Val dal Botsch; M–Val Trupchun/Val Müschauns; T–Val Tavrü, and Z–Val Zeznina/Macun. Mean values and ranges are presented for each transect line; n indicates the number of sampling sites. (DOC) [file pone.0271831.s009.doc]

**S3 Table. Soil characteristics in four transects in the SNP and its surroundings at site level.** B − Val dal Botsch; M – Val Trupchun/Val Müschauns; T – Val Tavrü, and Z – Val Zeznina/Macun. Mean values and ranges are presented for each valley; n indicates the number of sampling sites.

|  | Transect | | | |
| --- | --- | --- | --- | --- |
|  | B (n = 16) | M (n = 15) | T (n = 17) | Z (n = 17) |
| Organic layer (OL, cm) | 1.84 (0–10) | 0.83 (0–8) | 1.68 (0–8) | 0.97 (0–7) |
| Humic top soil layer (AH, cm) | 4.47 (3–5) | 5.40 (4–9) | 4.88 (3–5) | 5.59 (1–10= |
| Soil pH | 7.44 (7.22–8.10) | 5.83 (3.94–7.99) | 7.31 (6.96–7.62) | 4.29 (3.63–4.97) |
| Particle fractions (grain size): |  |  |  |  |
| Sand (%) | 57.4 (41.9–71.6) | 63.3 (16.2–77.5) | 52.5 (13.8–67.7) | 71.3 (66.9–80.5) |
| Clay (%) | 11.0 (2.0–24.1) | 9.4 (5.3–17.4) | 15.5 (4.3–34.4) | 6.3 (3.4–8.2) |
| Silt (%) | 31.6 (20.4–45.9) | 27.3 (13.8–73.1) | 32.0 (7.9–59.6) | 22.4 (16.2–26.9) |
| Total nitrogen content (%) | 0.43 (0.00–0.81) | 0.47 (0.03–1.33) | 0.65 (0.13–1.81) | 0.48 (0.13–1.32) |
| Total carbon content (%) | 14.54 (9.64–18.58) | 8.98 (2.64–28.12) | 14.86 (3.97–24.17) | 7.37 (2.36–24.09) |
| Organic carbon content (%) | 6.28 (0.14–10.85) | 7.28 (0.42–28.12) | 8.92 (1.76–21.66) | 7.37 (2.36–24.09) |
| Inorganic carbon content (%) | 8.27 (2.40–12.38) | 1.59 (0.00–11.58) | 5.94 (0.43–12.28) | 0.00 (0.00–0.00) |
| C/N-ratio | 37.29 (16.34–71.37) | 20.41 (11.84–48.46) | 29.45 (12.55–94.69) | 15.53 (12.98–21.45) |
