# Supplementary material for: Six groups of ground-dwelling arthropods show different diversity responses along elevational gradients in the Swiss Alps
Source: PLoS One. 2022 Jul 25;17(7):e0271831. doi: 10.1371/journal.pone.0271831 (PMC9312367; doi:10.1371/journal.pone.0271831)
Supplement: S4 Table — (DOC) [file pone.0271831.s010.doc]

**S4 Table. Individual abundance and number of species (in parenthesis) in different ground-dwelling arthropod groups recorded in each of four transect lines in the SNP and its surroundings.** B − Val dal Botsch; M – Val Trupchun/Val Müschauns; T – Val Tavrü, and Z – Val Zeznina/Macun.

|  | Transect | | | | |
| --- | --- | --- | --- | --- | --- |
|  | B | M | T | Z | Total |
| Spiders | 322 (32) | 267 (64) | 169 (34) | 208 (35) | 966 (86) |
| Millipedes | 659 (15) | 428 (16) | 1062 (12) | 591 (13) | 2740 (21) |
| Centipedes | 193 (12) | 404 (13) | 329 (15) | 232 (14) | 1158 (19) |
| Ants | 3208 (7) | 2683 (7) | 1112 (10) | 465 (6) | 7468 (14) |
| Ground beetles | 449 (15) | 201 (19) | 292 (19) | 464 (24) | 1406 (34) |
| Rove beetles | 95 (21) | 309 (46) | 114 (22) | 526 (50) | 1044 (74) |
