# Supplementary material for: Six groups of ground-dwelling arthropods show different diversity responses along elevational gradients in the Swiss Alps
Source: PLoS One. 2022 Jul 25;17(7):e0271831. doi: 10.1371/journal.pone.0271831 (PMC9312367; doi:10.1371/journal.pone.0271831)
Supplement: S12 Table — (DOCX) [file pone.0271831.s018.docx]

**S12 Table. Summary of the linear mixed models (LME) examining the effects of elevation (elevational bands), area of elevational bands, aspect, inclination, heat load, type of vegetation and substrate and soil characteristics including depth of organic layer, pH, C/N-ratio and clay content (%) on the number of individuals belonging to six arthropod groups.**

|  | **Number of individuals** | | | | | |
| --- | --- | --- | --- | --- | --- | --- |
|  | **Spiders ^3^** | **Millipedes ^1^** | **Centipedes ^3^** | **Ants ^3^** | **Ground beetles ^1^** | **Rove beetles ^1^** |
| Elevational band | F_1,23_ = 10.88, **P = 0.031** | F_1,25_ = 0.01, P = 0.94 | **F**_1,19_ = 4.81, **P = 0.041** | F_1,24_ = 30.49**, P <0.0001** | F_1,17_ = 3.06, P = 0.10 | F_1,21_ = 37.59, **P <0.0001** |
| Elevational band area ^1^ | **–** | F_1,25_ = 1.20, P = 0.28 | **–** | **–** | **–** | **–** |
| Aspect | F_6,23_ = 3.32**, P = 0.017** | F_6,25_ = 3.55**, P = 0.011** | F_6,19_ = 1.89, P = 0.14 | F_6,24_ = 3.70, **P = 0.010** | F_2,17_ = 0.82, P = 0.57 | F_6,21_ = 5.32**, P = 0.002** |
| Inclination ^2^ | **–** | **–** | **–** | **–** | F_1,17_ = 0.05, P = 0.82 | F_1,21_ = 2.02, P = 0.17 |
| Heat load ^3^ | F_1,23_ = 5.43, **P = 0.029** | F_1,25_ = 1.22, P = 0.28 | F_1,19_ = 1.41, P = 0.25 | F_1,24_ = 2.97, P = 0.10 | F_1,17_ = 1.42, P = 0.25 | **–** |
| Vegetation type | F_2,23_ = 6.55, **P = 0.006** | **–** | F_2,19_ = 1.16, P = 0.34 | F_2,24_ = 1.70, P = 0.20 | F_2,17_ = 6.52, **P = 0.008** | F_2,21_ = 1.73, P = 0.20 |
| Substrate type | **–** | **–** | F_2,19_ = 1.58, P = 0.23 | **–** | F_2,17_ = 2.37, P = 0.12 | F_2,21_ = 2.40, P = 0.12 |
| Organic layer ^3^ | **–** | **–** | **–** | **–** | F_1,17_ = 0.01, P = 0.96 | **–** |
| Soil pH ^3^ | F_1,23_ = 1.35, P = 0.26 | **–** | F_1,19_ = 8.98, **P = 0.007** | **–** | F_1,17_ = 9.52, **P = 0.007** | F_1,21_ = 12.44, **P = 0.002** |
| Soil C/N-ratio ^4^ | **–** | **–** | F_1,19_ = 0.94, P = 0.34 | **–** | F_1,17_ = 0.01, P = 0.91 | **–** |
| Soil clay content ^4^ | **–** | **–** | F_1,19_ = 3.74, P = 0.07 | **–** | F_1,17_ = 3.80, P = 0.07 | **–** |

Significant P-values (P < 0.05) are in bold

“–“ variable was excluded from the model due to the step-wise model reduction procedure

1 = log-transformed, 2 = sqrt-transformed, 3 = Tukey-transformed, 4 = arcsine-sqrt-transformed
