# Supplementary material for: Six groups of ground-dwelling arthropods show different diversity responses along elevational gradients in the Swiss Alps
Source: PLoS One. 2022 Jul 25;17(7):e0271831. doi: 10.1371/journal.pone.0271831 (PMC9312367; doi:10.1371/journal.pone.0271831)
Supplement: S13 Table — (DOCX) [file pone.0271831.s019.docx]

**S13 Table. Number of indicator species in each arthropod group for three vegetation types.** Only species for which the analysis revealed a significant IndVal value are shown, with species, whose IndVal is ≥ 70% shown in bold. The IndVal is given in square brackets. The number of species analysed for each arthropod group is given for comparison.

| **Vegetation type**  **Arthropod group** | **Number of species considered** | **Species with significant IndVal** | **Indicator species** |
| --- | --- | --- | --- |
|  |  |  |  |
| **Forest** |  |  |  |
| Spiders | 86 | 5 | *Robertus truncorum* [65], *Micaria aenea* [64], *Diplostyla concolor* [50], *Pardosa ferruginea* [50], *Trochosa ruricola* [50] |
| Millipedes | 21 | 6 | ***Polydesmus monticola*** [76], ***Chordeumatida* sp.** [73], *Cylindroiulus zinalensis* [64], *Atractosoma meridionale* [61], *Iulogona tirolensis* [56], *Cylindroiulus tirolensis* [54] |
| Centipedes | 19 | 2 | *Lithobius tenebrosus* [64], *Eupolybothrus tridentinus* [61] |
| Ants | 14 | 3 | ***Formica lugubris*** [76], *Formica aquilonia* [50], *Myrmica lobicornis* [50] |
| Ground beetles | 34 | 5 | ***Calathus micropterus*** [76], ***Pterostichus unctulatus*** [75], *Cychrus attenuatus* [65], *Trichotichnus laevicollis* [60], *Pterostichus jurinei* [59], |
| Rove beetles | 74 | 8 | ***Zyras humeralis*** [75], ***Lordithon exoletus*** [71], *Liogluta wuesthoffi* [67], *Quedius ochropterus* [66], *Quedius paradisianus* [62], *Tachinus elongatus* [61], *Tachinus laticollis* [50], *Tachinus proximus* [50] |
|  |  |  |  |
| **Grassland** |  |  |  |
| Ground beetles | 34 | 1 | *Cymindis vaporariorum* [59] |
|  |  |  |  |
| **Patchy veg.** |  |  |  |
| Spiders | 86 | 2 | *Xysticus lanio* [64], *Drassodes heeri* [56] |
| Millipedes | 21 | 1 | ***Leptoiulus helveticus*** [75] |
| Centipedes | 19 | 2 | ***Lithobius glacialis*** [83], *Lithobius* sp. [67] |
|  |  |  |  |
